# Differences in Disrupted Dynamic Functional Network Connectivity Among Children, Adolescents, and Adults With Attention Deficit/Hyperactivity Disorder: A Resting-State fMRI Study

**DOI:** 10.3389/fnhum.2021.697696

**Published:** 2021-10-05

**Authors:** Elijah Agoalikum, Benjamin Klugah-Brown, Hang Yang, Pan Wang, Shruti Varshney, Bochao Niu, Bharat Biswal

**Affiliations:** ^1^MOE Key Laboratory for Neuroinformation, School of Life Sciences and Technology, The Clinical Hospital of Chengdu Brain Science Institute, University of Electronic Science and Technology of China, Chengdu, China; ^2^Department of Biomedical Engineering, New Jersey Institute of Technology, Newark, NJ, United States

**Keywords:** ADHD, brain connectivity, dynamic functional brain network, fMRI, brain networks and dynamic connectivity, resting state—fMRI

## Abstract

Attention deficit hyperactivity disorder (ADHD) is one of the most widespread mental disorders and often persists from childhood to adulthood, and its symptoms vary with age. In this study, we aim to determine the disrupted dynamic functional network connectivity differences in adult, adolescent, and child ADHD using resting-state functional magnetic resonance imaging (rs-fMRI) data consisting of 35 children (8.64 ± 0.81 years), 40 adolescents (14.11 ± 1.83 years), and 39 adults (31.59 ± 10.13 years). We hypothesized that functional connectivity is time-varying and that there are within- and between-network connectivity differences among the three age groups. Nine functional networks were identified using group ICA, and three FC-states were recognized based on their dynamic functional network connectivity (dFNC) pattern. Fraction of time, mean dwell time, transition probability, degree-in, and degree-out were calculated to measure the state dynamics. Higher-order networks including the DMN, SN, and FPN, and lower-order networks comprising the SMN, VN, SC, and AUD were frequently distributed across all states and were found to show connectivity differences among the three age groups. Our findings imply abnormal dynamic interactions and dysconnectivity associated with different ADHD, and these abnormalities differ between the three ADHD age groups. Given the dFNC differences between the three groups in the current study, our work further provides new insights into the mechanism subserved by age difference in the pathophysiology of ADHD and may set the grounds for future case-control studies in the individual age groups, as well as serving as a guide in the development of treatment strategies to target these specific networks in each age group.

## Introduction

Attention deficit hyperactivity disorder (ADHD) is one of the most common mental disorders worldwide, characterized by inattentive, hyperactive, or impulsive behaviors (American Psychiatric Association., 2000). ADHD mostly affects children, but often persists to adulthood (Danielson et al., 2018). The Diagnostic and Statistical Manual of Mental Disorders-IV (DSM-IV) classified ADHD into 3 sub-types, namely; hyperactive/impulsive (HI), inattentive (IA), and combined (C) type. ADHD prevalence in children, adolescents, and adults is 9.5, 11.4, and 4.4%, respectively (Gimpel and Kuhn, 2000; Barbaresi et al., 2002; Kessler et al., 2006; Wolraich et al., 2011), with its symptoms varying from one age group to the other (Katragadda and Schubiner, 2007).

Functional magnetic resonance imaging (fMRI) has become a popular technique for studying brain diseases or disorders such as ADHD. The majority of these ADHD fMRI studies have followed a task-based approach, aiming to examine how brain function may be modulated by group status during cognitive task performance. This approach is designed to isolate specific cognitive processes that may be linked to or modified by ADHD symptoms or treatment. In recent times, however, there has been an overwhelming interest in an alternative method called resting-state functional magnetic resonance imaging (rs-fMRI). The term “resting state” is misleading because the brain is never at rest (Stark and Squire, 2001; Raichle, 2006). It is often used to denote a task-free procedure where participants are asked to lie still in a scanner, with their eyes either opened or closed, and not think about anything specific. Resting-state fMRI gives a measure of brain neurophysiology that is not dependent on task-directed cognitive processes. Moreover, the discovery of the default mode network of brain structures, which is said to be active during the resting state and show dynamic negative correlations with task-related regions, has opened up new areas of investigation (Raichle et al., 2001; Greicius et al., 2003) and has raised interesting questions with regards to abnormal patterns of brain activation in patients with ADHD.

Under resting-state conditions, intrinsic networks obtained from rs-fMRI correlate with low frequency BOLD signal fluctuations between regions of the brain (Biswal et al., 1995; Fox and Raichle, 2007; Khundrakpam et al., 2016). It has been demonstrated that the human brain is functionally organized into a hierarchy of large-scale connectivity networks (Meunier et al., 2009). Abnormal functional connectivity (FC) within the default mode, executive control, salience, and attention-related networks were observed in ADHD patients (Sidlauskaite et al., 2016; Bos et al., 2017). These networks are said to be associated with symptoms of ADHD, such as impairment of executive function processing and distractibility (Francx et al., 2015; Zhao et al., 2017). Significant differences were also observed between child and adolescent ADHD patients within the default mode and frontoparietal networks (Park et al., 2016), which are also said to be highly associated with ADHD symptoms (Buckner et al., 2008; Andrews-Hanna, 2012; Ptak, 2012). These and several other studies have found significant differences both between ADHD patients and healthy control subjects and also among ADHD patient groups, but most of these studies are based on the assumption that FC is static throughout the whole scan time and, therefore, calculate FC using the entire time course. Even though static functional network connectivity (sFNC) has been used to successfully determine brain abnormalities in ADHD and other neurological diseases, it has ignored the fact that different neural activities can occur at different points in time.

Having proven that FC of the resting brain is indeed dynamic (Fox et al., 2005), methods have been in development since 2010 to depict the time-varying network connectivity in rs-fMRI and to capture the network activity of the brain in more detail (Chang and Glover, 2010; Sakoǧlu et al., 2010).

In recent times, some researchers have observed time-varying connectivity patterns among intrinsic networks in mental disorders, such as schizophrenia and bipolar disorder, that cannot be detected using sFNC (Damaraju et al., 2014; Rashid et al., 2014). Dynamic functional network connectivity (dFNC) has yielded fascinating results in several brain disorders, showing the within and between network disconnections that may be unknown or uncertain (Damaraju et al., 2014; de Lacy et al., 2017; de Lacy and Calhoun, 2018). Previous works using task-based regions of interest suggest lagging strength in frontal-parietal-striatal-cerebellar connections in ADHD, with implications mainly in the frontoparietal, ventral attention, and default mode networks (Cortese et al., 2012; Hart et al., 2012, 2013).

Even though dFNC has been employed to study the differences between healthy control subjects and patients in several mental disorders including ADHD, no study has employed dFNC to access network connectivity differences between child, adolescent, and adult ADHD patients. Given that ADHD symptoms vary among the three age groups, identifying network disruptions specific to each age group will provide new insights into the pathophysiology of ADHD and may set the grounds for future case-control studies in the individual age groups, as well as serving as a guide in the development of treatment strategies to target these specific networks in each age group. We, therefore, performed dFNC using group independent component analysis (GICA), sliding window correlation, and K-means clustering to explore network connectivity differences in ADHD between these three age groups. We hypothesized that dFNC can capture the time-varying characteristics of fMRI data perculiar to the three ADHD age groups.

## Materials and Methods

### Data Acquisition

Unprocessed resting-state fMRI data of ADHD patients (158 subjects) were obtained from the New York University Child Study Center for the ADHD-200 Global Competition and UCLA dataset (Bilder et al., 2016). The NYU dataset is made up of 45 child ADHD patients, and 73 adolescent ADHD patients. The UCLA dataset is made up of 40 adult ADHD patients. Both datasets are made openly to researchers online. All participants used in the current study were diagnosed with ADHD and their symptom scores have been used for the correlation analysis in the current study. Detailed information about the subjects can be found in Table 1.

**TABLE 1:** Data demographics.

ADHD group	Adults (*n* = 39)	Adolescents (*n* = 40)	Adolescents (*n* = 40)	*P*-value
Gender (M/F) 0.026224	(20/19)	(31/9)	(26/9)	
Age (years)	31.59 ± 10.13	14.11 ± 1.83	8.64 ± 0.81	<0.00001
Data range (years)	21–50	11.41–17.61	7.24–9.98	
OA score	63.49 ± 4.99	70.18 ± 46	70.74 ± 7.81	
H score	31.57 ± 4.63	65.95 ± 11.89	66.69 ± 12.69	
IA score	35.77 ± 2.78	68.88 ± 9.16	69.89 ± 8.87	

*One-ANOVA was used to determine group differences. OA, Overall severity; H, Hyperactivity severity; IA, Inattentive severity.*

For the adult dataset, MRI data were acquired on one of two 3T Siemens Trio scanners, located at the Ahmanson-Lovelace Brain Mapping Center (Siemens version syngo MR B15) and the Staglin Center for Cognitive Neuroscience (Siemens version syngo MR B17) at UCLA. Functional MRI data were collected using a T2^∗^-weighted echo-planar imaging (EPI) sequence with the following parameters: slice thickness = 4 mm, slices = 34, TR = 2 s, TE = 30 ms, flip angle = 90°, matrix 64 × 64, FOV = 192 mm. Additionally, a T2-weighted matched-bandwidth high-resolution anatomical scan (with the same slice prescription as the fMRI scan) and MPRAGE were collected. The parameters for the high-resolution scan were: 4 mm slices, TR/TE = 5,000/34 ms, 4 averages, matrix = 128 × 128, 90-degree flip angle. The parameters for MPRAGE were the following: TR = 1.9 s, TE = 2.26 ms, FOV = 250 mm, matrix = 256 × 256, sagittal plane, slice thickness = 1 mm, 176 slices. For the child and adolescent datasets, MRI data were obtained using Siemens Magnetom Allegra Syngo Mr 2004a. FMRI data were collected using an echo-planar imaging sequence with the following parameters: slice thickness: 4 mm, Slices: 33, TR: 2,000 ms, TE: 15 ms, Rotation = 90°, FoV phase: 80.0%, FoV read = 240 mm. In addition, T1-weighted images were acquired using the following parameters: Slice thickness = 1.33 ms, TR = 2,530,ms, TE = 3.25,ms, rotation = 0 degrees, FoV phase = 100.0%, FoV read = 256 mm.

### Data Preprocessing

For the adult dataset, the first 2 time points were removed, leaving final time points of 150. For the pediatric (adolescent and child) datasets, the first 26 time points were removed to ensure that all the data have equal time points since the time courses are used in the dFNC calculations. The same preprocessing steps were done for all subjects, including slice time correction, realignment, co-registration of T1 images to corresponding functional images, segmentation, normalization by Diffeomorphic Anatomical Registration using Exponentiated Lie algebra (DARTEL) (Ashburner, 2007), and resampling to 3 × 3 × 3 mm voxels, nuisance covariates regression using Friston 24 (Friston et al., 1996), and spatial smoothing with a 6 mm full width half maximum (FWHM) Gaussian kernel. Pediatric and adult datasets were preprocessed separately to ensure that the right template is generated for normalization. Subjects with a maximum translation > 2 mm or rotation > 2° were excluded from further analysis, leaving a total of 114 subjects. The final data used for further analysis included 35 children (8.64 ± 0.81 years), 40 adolescents (14.11 ± 1.83 years), and 39 adults (31.59 ± 10.13 years). All preprocessing steps were performed using the data processing assistant for resting-state fMRI, advanced edition (DPARSFA) in the DPABI toolbox (Yan et al., 2016).

### Group Spatial Independent Component Analysis

Data were decomposed into functional networks using a group-level spatial ICA as implemented in the GIFT toolbox.^1^ A relatively high model order with 60 components was performed using the Infomax algorithm with a best-run selection from 10 randomly initialized analyses to achieve a functional parcellation of refined cortical and subcortical components corresponding to known anatomical and functional segmentations (Himberg et al., 2004; Li et al., 2007; de Lacy and Calhoun, 2018). To make sure that all components selected were intrinsic component networks (ICNs), sorting was performed using a combination of visual inspection and quantitative metrics. For each of the 60 components, spectral metrics of (1) fractional amplitude of low-frequency fluctuations (fALFF) and (2) dynamic range were generated. Generally, independent components representing brain networks are said to have higher values in these spectral metrics, whereas noise components are said to have lower values (Allen et al., 2011, 2012). Hence, we checked the spectral metrics of each component, and only components with high values in these spectral metrics were retained for further scrutiny. Components were also visually inspected, and their peak coordinates were used to determine their correspondence with gray matter. Components with poor overlap with the cerebral gray matter or low spectral metrics were discarded. The remaining 50 components represented the intrinsic networks (INs) used in this study.

### dFNC Analysis

The selected components underwent additional post-processing (linear detrending, despiking, and low-pass filtering with a high-frequency cutoff at 0.15 Hz) to remove any remaining sources of noise. DFNC was estimated based on the sliding window approach. Based on previous studies (Allen et al., 2014; Klugah-Brown et al., 2019; Sanfratello et al., 2019), we selected a window width size of 22 TRs = 44 s, and sliding steps of 1 TR, resulting in 128 windows. This was obtained for all 114 subjects to give a total of 14,592 instances (114 subjects × 128 windows). For each window, FNC was estimated between ICNs from a regularized inverse covariance matrix using the graphical LASSO method (Friedman et al., 2008). An L1 norm was placed on the inverse covariance matrix to promote sparsity, and the regularization parameter (lambda) was optimized for each subject. The dFNC values were Fisher-Z transformed. In brief, the graphical LASSO method is a method used for learning the structure in an undirected Gaussian graphical model, which uses L1 regularization in controlling the number of zeros in the precision matrix Θ = Σ^–1^. Kindly refer to Meinshausen and Bühlmann (2006); Yuan and Lin (2007), and Banerjee et al. (2008) for more information.

K-means clustering was used to cluster all dFNC windows based on the correlation distance. Clustering numbers from 2 to 10 were selected, representing cluster states. For each k, the clustering algorithm was repeated 500 times to increase the chances of escaping local minima, with random initialization used to obtain the state cluster centroids. The optimal number of clusters was estimated using the elbow criterion and silhouette algorithms. An optimal K = 3 was obtained using these two methods. Also, subjects were well distributed among these three clusters, which is better for pattern evaluation. Only the selected 50 ICNs were used in the clustering, resulting in a total of [50 × (50–1)]/2 = 1,225 features. These features were then used for the dynamic FNC analysis between the three groups.

**FIGURE 1:**
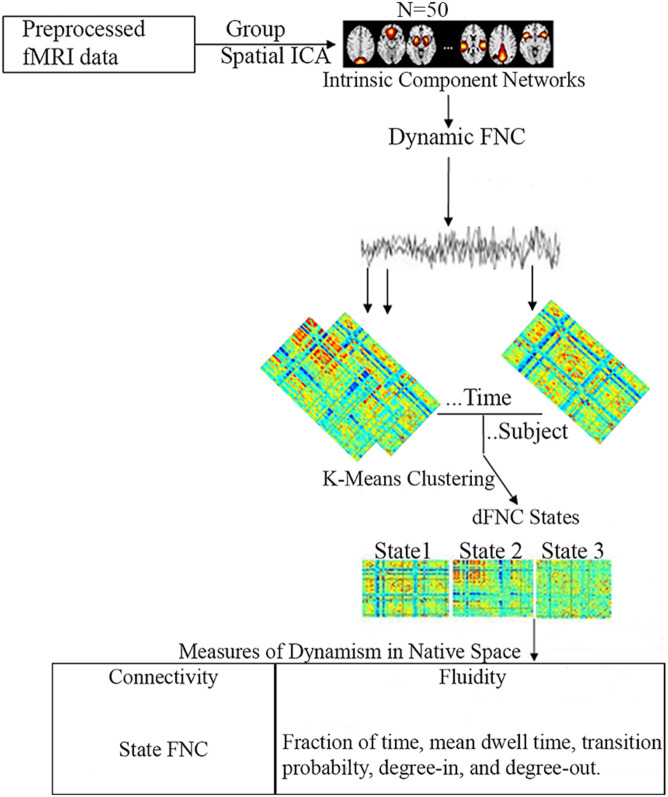
Schematic diagram of the analysis pipeline. Rs-fMRI data was preprocessed and subjected to spatial group ICA resulting in 50 intrinsic component networks. Static FNC was then estimated. For dFNC, the sliding window approach was adopted and clustered using K-means to obtain 3 cluster states. Based on these three cluster states, five measures of dynamism were computed in native space.

Functional network connectivity, fraction of time, mean dwell time, transition probability, degree-in, and degree-out were compared between the three age groups in each state. Correlation analyses were also performed to determine the impact of age, overall ADHD severity, hyperactivity severity, and inattentive severity on fraction of time and mean dwell time of each age group in each cluster state. All statistical analyses were performed using MATLAB (Mathworks Inc., United States). Age, gender, and mean framewise displacement (mean FD) were used as covariates for statistical analyses. Furthermore, given that our data was obtained from different sites, and several studies have shown the effect of multi-site in different ADHD age groups (Hong et al., 2017; Zhou et al., 2019), we regressed out the effect of site in our analyses. In brief, the NYU dataset (consisting of the pediatric dataset) was represented as site one (1), whiles the UCLA dataset (consisting of the adult dataset) was represented as site two (2). Thus, each subject in the NYU and UCLA datasets were labeled 1 and 2, respectively, and used as covariates in our statistical analysis to regress out site effects. False discovery rate (FDR) was used for multiple comparison corrections. Figure 1 shows the schematic diagram of the analysis pipeline.

## Results

### Spatial ICA and ICNs

Spatial maps of the ICNs and their time courses were decomposed using GICA. The selected 50 ICNs were further categorized into nine networks based on their anatomical and functional properties, including the sensorimotor network (SMN), visual network (VN), default-mode network (DMN), central executive network (CEN), cerebellum network (CBN), subcortical network (SC), auditory network (AUD), frontoparietal network (FPN), and salience network (SN). The identified ICNs with their activation peaks primarily fell on the gray matter (Supplementary Figure 2).

### Dynamic FNC States

**FIGURE 2:**
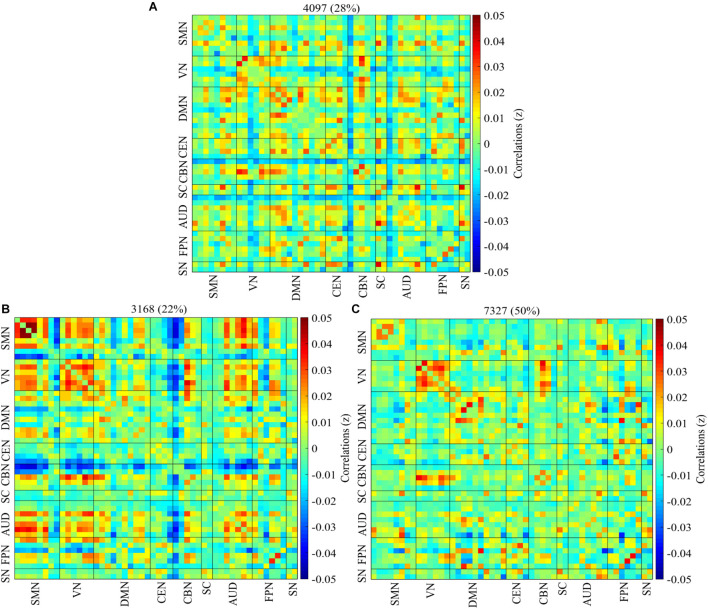
Percentage of occurrence. The median of all subjects together with the total number and percentage of occurrences are displayed in each state. **(A–C)** Represent states 1–3. The connectivity pattern varies among the three cluster states with state 2 showing more connectivity than states 1 and 3.

Three reoccurring dFNC states over time were identified using K-means clustering and the cluster centroid of each dFNC state is shown in Figure 2. All three states showed positive connectivity within the VN. States 1 and 3 showed positivity connectivity within the DMN, with state 1 distinguishing itself with negative connectivity between some ICNS of the CBN, AUD, and VN with other networks. States 2 and 3 showed positive connectivity within the VN and SMN, with state 2 showing strong positive connectivity within these two networks than state 3, but some ICNs showed strong negative connectivity within these two networks. State 2 also showed strong positive connectivity between the SMN and VN, with the CBN showing antagonism with other networks in this state.

### Group Differences in dFNC States

To find out if there are significant dFNC differences between the three age groups, two-sample *t*-tests were done between 1. Adolescents vs. children 2. Adults vs. children 3. Adults vs. adolescents.

#### Adolescents vs. Children

**FIGURE 3:**
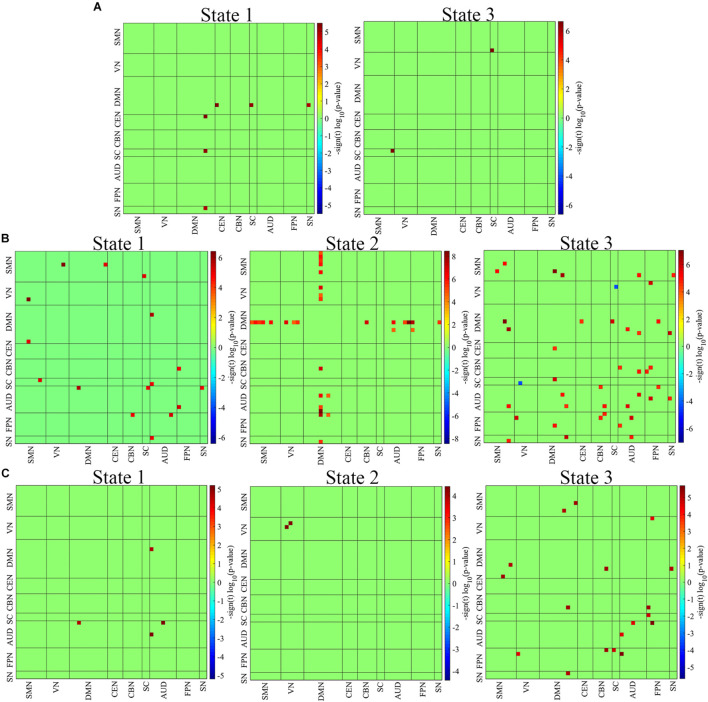
Group differences among the three age groups. **(A)** Adolescent vs. Child ADHD patients. **(B)** Adult vs. Child ADHD patients. **(C)** Adult vs. Adolescent ADHD patients. Two out of the three clusters showed significant differences between child and adolescent ADHD patients, whiles all three clusters showed significant differences between child/adolescent and adult ADHD patients (*P* < 0.01, FDR corrected). The red squares indicate increased network connectivity whiles the blue squares indicate decreased network connectivity.

Figure 3A demonstrates significant differences (*p* < 0.01, FDR corrected) between adolescent and child ADHD groups. Relative to child ADHD patients, adolescent ADHD patients showed increased network connectivity between the DMN and CEN, DMN and SC, and DMN and SN in state 1. Compared to Child ADHD patients, adolescent ADHD patients exhibited increased network connectivity between the SMN and SC in state 2.

#### Adults vs. Children

The results of the two-sample *t*-test between adults and children are shown in Figure 3B. All three clusters were found to show significant differences between the two groups (*p* < 0.01, FDR corrected). Relative to child ADHD patients, adult ADHD patients showed increased network connectivity between the SMN and VN, SMN and DMN, SMN and SC, and between the AUD and SC, AUD and DMN, AUD and SN, AUD and FPN, and FPN and CBN in state 1. In state 2, the connectivity pattern changed, with increased network connectivity between the DMN and SMN, DMN and VN, DMN and CBN, DMN and AUD, DMN and FPN, and DMN and SN in adult ADHD patients relative to child ADHD patients. The connectivity pattern again changed in state 3 with more network connectivity differences between the two groups. The DMN showed increased connectivity with the CEN, SC, AUD, FPN, and SN, whiles the VN showed decreased connectivity with the SC in adult ADHD patients relative to child ADHD patients. Compared to the child group, increased connectivity was found within the SMN and AUD, and between the SMN and AUD, SMN and SN, AUD and CBN, AUD and FPN, and AUD and SN in the adult group in state 3.

#### Adults vs. Adolescents

All three cluster states showed significant differences between the 2 groups (*p* < 0.01, FDR corrected) (Figure 3C). In state 1, adult ADHD patients showed increased network connectivity within the AUD, and between the AUD and DMN relative to their adolescent counterparts. Compared to adolescent ADHD patients, increased network connectivity was found within the VN in adult ADHD patients in state 2. Relative to adolescents ADHD patients, adult ADHD patients again showed increased network connectivity within the AUD, between the DMN and SMN, DMN and CBN, and between the DMN and SN in state 3. Also, in state 3, increased network connectivity was observed between the FPN and VN, FPN and CBN, and FPN and SC in adult ADHD patients relative to adolescent ADHD patients. The DMN connectivity difference between the two groups varies across the 3 states. Interestingly, the FPN showed no significant connectivity differences between the 2 groups in states 1 and 2 but showed significant connectivity differences with the VN, CBN, and SC in state 3.

### Fluidity Measures

**FIGURE 4:**
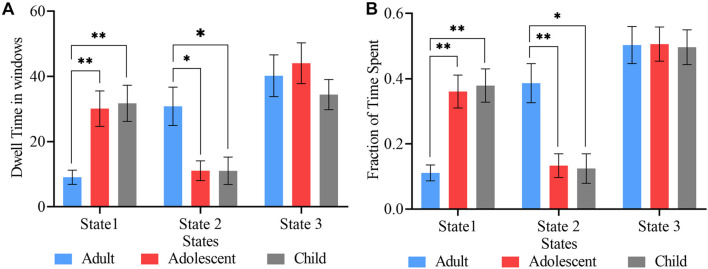
State vectors for temporal analysis. **(A)** Mean dwell times in the three cluster states. **(B)** Fraction of time spent by each group in the three states. The blue, red, and ash bars represent adult, adolescent, and child ADHD patients, respectively. Asterisk indicates *P* < 0.05, FDR corrected and two asterisks indicate *P* < 0.001, FDR corrected.

The mean dwell time, which is the mean time spent in one state before moving to the next state, was compared between the three age groups using two-sample *t*-tests (Figure 4A). In state 1, the child group showed the highest mean dwell time, whiles the adult and adolescent groups showed the highest mean dwell times in states 2 and 3, respectively. However, significant differences were observed between only adult and adolescent, and adult and child ADHD pairs in states 1 and 2 (FDR corrected), with no significant mean dwell time differences between adolescent and child ADHD patients in all three cluster states.

The proportion of time each subject stayed in each state within the whole scan duration was defined as the fraction of time in that state. Figure 4B shows the fraction of time spent in each state over the whole time series. Two-sample *t*-tests were performed to determine the differences in fraction of time between the three age groups. Significant differences in fraction of time were found between adult and adolescent, and adult and child pairs in only states 1 and 2 (FDR corrected). There was no significant fraction of time difference between adolescent and child ADHD pair in any of the three cluster states.

**FIGURE 5:**
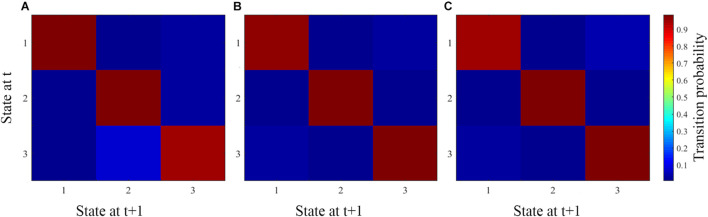
The average transition matrix and transition probabilities of each age group. **(A–C)** Represent the average matrix for adult, adolescent, and child ADHD patients, respectively.

The average transition matrices and transition probabilities of each age group are shown in Figure 5, which represent the probability of changing from one state to the other. The red squares along the main diagonals represent a high probability of staying in a particular state, hence, the deeper the red square, the higher the probability of staying in a particular state. The blue squares represent the probability of moving between states, hence, the lighter the blue square, the higher the probability for subjects to move between states. The light blue square in the column of state 2 and the row of state 3 (Figure 5A), indicates a high probability of adult subjects moving between these two states, which is evident in the adult mean dwell time and fraction of time spent in these two states. Likewise, adolescent and child patients have high probabilities of moving between states 1 and 3 (Figures 5B,C), which is evident in the mean dwell time and the fraction of time they spent in these two states.

**FIGURE 6:**
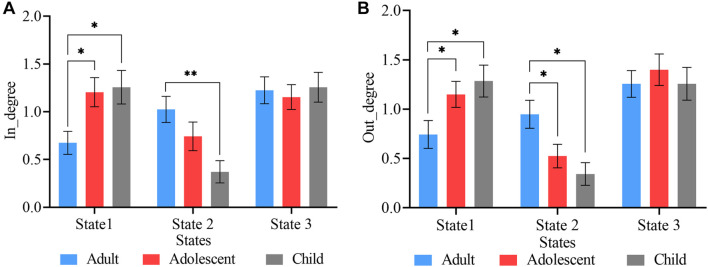
State vectors degree of transitions. **(A)** Frequency of transitions into each state. State 1 showed significant differences in both adult vs. adolescent, and adult vs. child pairs, whereas, state 2 showed significant differences between only adult vs. child ADHD patients. **(B)** Frequency of transitions out of each state. States 1 and 2 showed significant differences between both adult vs. adolescent, and adult vs. child pairs. Asterisk indicates significant states (*p* < 0.05, FDR corrected), two asterisks indicate significant differences with threshold *p* < 0.001, FDR corrected.

The total frequency of transitions from other states into a particular state (referred to as degree-in) (Figure 6A) and the total frequency of transitions from a particular state into other states (referred to as degree-out) (Figure 6B) was calculated for the three groups in all three states and two-sample *t*-tests were used to determine the differences between the three groups. Significant degree-in differences were found between adult and adolescent, and adult and child pairs in state 1 (*P* < 0.05, FDR corrected), whiles state 2 shows significant differences between only adult and child pair (*P* < 0.001, FDR corrected) (Figure 6A). However, both states 1 and 2 showed significant degree-out differences between adult and adolescent, and adult and child ADHD pairs (Figure 6B). No significant differences were found between adolescent and child ADHD patients in both degree-in and degree-out.

### Correlation Analyses

**FIGURE 7:**
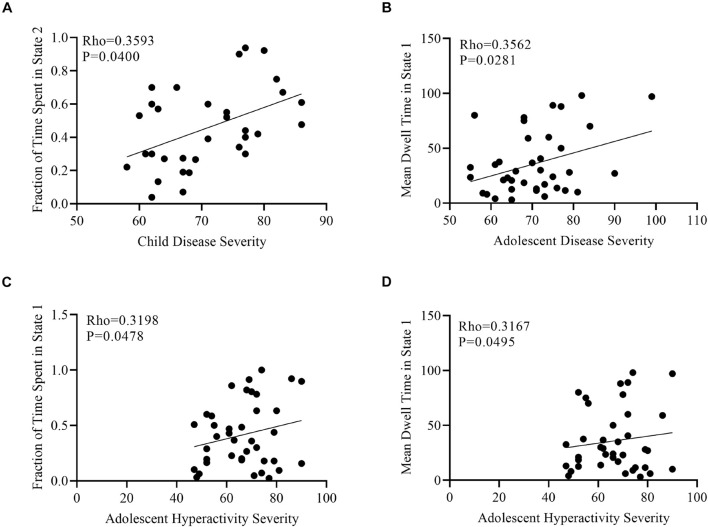
Scatter plots of the correlation analyses. **(A)** Positive correlation between fraction of time spent in state 2 and child disease severity. **(B)** Positive correlation between mean dwell time in state 2 and adolescent disease severity. **(C)** Positive correlation between fraction of time spent in state 1 and adolescent hyperactivity severity. **(D)** Positive correlation between mean dwell time in state 1 and adolescent hyperactivity severity.

Correlation analyses were performed to determine the impact of age, overall ADHD severity, hyperactivity severity, and inattentive severity on fraction of time and mean dwell time for each age group in each cluster state using gender and age as covariates. No significant correlations were found between fractions of time and mean dwell time for the above-mentioned measures in all three states in adult ADHD patients. A significant positive correlation was found between overall disease severity and fraction of time in state 2 in the child group (Figure 7A). In the adolescent group, mean dwell time in state 1 was positively correlated with overall disease severity and hyperactivity severity (Figures 7B,D), whiles faction of time in this same state was positively correlated with only hyperactivity severity (Figure 7C).

## Discussion

In this study, we investigated time-varying network connectivity patterns and network disruptions in child, adolescent, and adult ADHD patients using ICA, sliding windows, and K-means clustering. Our analysis revealed the following results: (1) unique state network alterations between the resting-state networks were found in all the three groups of ADHD patients with disruption occurring mainly in lower-order functional networks including SMN, AUD, VN, and CBN, while higher-order networks (DMN, SN, CEN, and FPN) showed rather sparse and low connectivity; (2) changes in state vectors as a measure of dynamic changes were obtained for the three groups including mean dwell time, fraction of time spent, number of transitions across states, and total transition measure by the degree in and out of state; (3) mean dwell time was positively correlated with overall disease severity and hyperactivity severity in only the adolescent group, whiles fraction of time was positively correlated with overall severity in the child group and hyperactivity severity in the adolescent group.

To the best of our knowledge, our work is the first to explore dFNC of the three ADHD age groups, adding to the increasing literature on the evidence of dFNC (Chang and Glover, 2010; Sakoǧlu et al., 2010; Allen et al., 2014; Damaraju et al., 2014; Rashid et al., 2014; Klugah-Brown et al., 2019) and how it can capture disruption among ADHD patient across different age ranges. The implications of functional interconnections between resting-state networks have gained full attention over the years and have become a robust tool to investigate brain disorders (find extensive review and meta-analysis in Cortese et al., 2021). However, these studies rely on the assumption that the FC derived static throughout scanning time. In contrast to this assumption, it’s been since 2010 that the brain states are more dynamic across time and that a time-varying approach may provide a better view of this phenomenon (Chang and Glover, 2010; Sakoǧlu et al., 2010), as well as being able to capture the dynamic connectivity patterns across time.

Three reoccurring states were found using K means clustering, and the connectivity patterns were relatively similar across the groups in all three states (Supplementary Figure 3). We observed varying connectivity patterns across states with each state exhibiting different occurrences denoted by a percentage of the total instances for all groups and subjects (Figure 2). The measurement taken over longer time windows summarize anatomical connectivity, which reflects RSNs. However, measurements taken over shorter window times accentuate the small departure from the RSN pattern, forming new functional networks from different nodes for a short period and then returning to the RSN pattern. Even though certain functional networks are often repeated in time, their exact organization or arrangement at a particular point depends on the part of the dynamic repertoire being explored (Deco et al., 2011), this results in the differences in the dynamic patterns of the three cluster states. The connectivity patterns within and between lower-order networks was in contrast with disruption expected suggested to occur within and between FPN and other higher-order networks in ADHD (Gao et al., 2019) networks including DMN, FPN, and CEN, this suggests that although altered differences occurred, it did not represent case-control patterns but rather reflected age-driven association among the ADHD groups. The higher-order networks on the contrary showed a rather weaker hyperconnectivity among the groups for all three states. The hyperconnectivity found within and between the SMN, VN, and AUD in the highly connected state 2, highly synchronous patterns were different, with generally more sparse connections in state 3 within the DMN, and between the DMN and FPN compared to state 2. The DMN which is involved in self-referencing and abrupt inattentiveness (Buckner et al., 2008; Fox et al., 2015; Bozhilova et al., 2018) exhibited weaker within and between connectivity is consistent with previous studies in ADHD reflected declining capacity to integrate within network activities similar to those found in case-control groups (Lin and Gau, 2015). In addition, the relatively low connectivity between the FPN and DMN resonates with Castellanos et al. (2008) study in which using resting-state fMRI they showed an anticorrelation between the above networks and suggested that the observed pattern reflected a decline in the attention processes in ADHD which is subserved by the higher-order network in FPN.

On the group comparing of the k-mean clusters and the group level connectivity difference, we found different state patterns peculiar to each group. Supplementary Figure 3 shows the group-specific centroids indication the number of subjects contributing to each state. Following the clusters for each state, the group spatial group differences were generally hyperconnected, Figure 3A demonstrates two significant states between adolescents and child ADHD with increased connectivity between the DMN and CEN, SC, and SN. As indicated in the above paragraph, the inattention modulated by DMN is linked with the FPN, however, in both states, the connectivity with the CEN only re-echoed the control networks which are altered involving SN and SC. Inattention has been suggested to be increased in DMN with lower-order networks such as the SC in a task-based study (Oldehinkel et al., 2016) which is parallel with our result. Figure 3B shows connectivity differences between adult and child ADHD, in all three states we found the DMN hyperconnection with yet the lower-order networks including AUD and SMN, and also between the lower-order network (SC, VN), altered activation in the occipital regions and disconnections between the occipital cortex and frontal cortex in child and adolescent ADHD patients has been reported in previous studies (Mazaheri et al., 2010; Kröger et al., 2014). Disruptions of the VN have also been reported in ADHD patients (Benli et al., 2018), indicating that the VN plays an important role in ADHD. In state 2, the DMN connected with the FPN, AUD, and within the DMN, which is evident in the task-positive network relating to disrupted attention maintenance, and signifying inattention was revealed in the following review (Luman et al., 2010). Relative to adolescent ADHD patients, increased connectivity was found within the VN in adult ADHD patients. In state 3, Adult ADHD showed increased connectivity between the SMN and DMN relative to child ADHD patients. The connectivity pattern was similar to the state but had a widespread connection between the lower-order networks. Both DMN and FPN were also present, however, there was no connection between them, which is in contrast with the previous notion that these networks provided the core role in the attention and self-referential system in children and adolescents (Houck et al., 2011). For adults and adolescents, we also found all the three states were significant with less connectivity within each state, Figure 3C. All connections were in the low-order networks; AUD and VN, in states 1 and 2, respectively. State 3 expressing comparatively more connectivity and was between DMN and the lower-order networks. The phenomenon is similar to those found in the other age groups, however, adults compared to adolescents seem to have a less significant connection across all networks. This may suggest that the transition from adolescence to adulthood had little influence on the overall connectivity pattern in ADHD. In brief for the spatial connectivity difference between each pair of groups, the DMN representing a higher-order network was disrupted. Also, FPN connectivity was most evident between the adolescent and children relative to adults and other groups. This pattern of connectivity suggests that DMN or self-referential modulated inattention in all groups. thus, the DMN has been reported to be disrupted in several ADHD studies (Cortese et al., 2012; Hart et al., 2013; Park et al., 2016; Qian et al., 2019), and significant connectivity differences across the three age groups found in the current study completed the previous studies.

In our state vector analysis, several temporal measures were captured across all states for each group. Firstly, both the dwell time and fraction of time showed significance between each pair of groups in states 1 and 2 except state 3 Figure 4, which reflected correspondence with the spatial results in which for each paired group the connectivity was more in tasks negative network (DMN) and signifies that mental state was not fully represented over all states. Similarly, the likelihood of transition among states showed a corresponding temporal pattern as shown in Figure 5. In addition, to further characterize the temporal dynamics with the disease scores, we performed a correlation between each score and the transition vectors. As displayed in Figure 7, the adolescent group disease severity and hyperactivity positively correlated with state 1 vectors (mean dwell time and a fraction of time spent), suggesting that duration of occupied state corresponded to disease, and as the mental state changes the severity increases. Also, child disease severity exhibited a positive relationship with the proportion of time spent in state 2. Adults group showed no significance for all measures across groups in each state. These finding implied that the disruption in children and adolescents were more prevalent and showed more dynamic relations relative to adults. Although not clear, we posit that adult ADHD is reduced, reflecting symptom decline in this age group (Katragadda and Schubiner, 2007; Volkow and Swanson, 2011; McCarthy et al., 2012).

Although we showed both temporal and spatial dFNC among the three groups, the result should be interpreted carefully. Firstly, the sample size was a limitation in this study, as connectivity tend to be more stable with an increasing number of participants, we were not able to investigate whether our relatively small sample influence the result presented, we suggest that future studies use larger sample sizes to determine the dFNC differences between the three ADHD age groups. Secondly, albeit the control of sex variable in the individual group analysis, the male to female ratio in the adult dataset was not the same as relative to child and adolescent datasets, which may interfere with brain state, future studies may consider recruiting more samples to include balanced sex ratio.

In sum, this study investigated the temporal and spatial dynamics of ADHD patients. Nine networks were identified using group ICA. Higher-order networks including the DMN and FPN and lower-order networks comprising the SMN, VN, and AUD were frequently distributed across all states and were connected within and between networks. We also found significant differences in measures such as mean dwell time, fraction of time, degree-in, and degree-out among the three age groups. Generally, all groups did not make full significant temporal transitions, only states 1 and 2 exhibited dynamic variability among the three groups. Our findings imply abnormal dynamic interactions and disconnectivity associated with ADHD. However, these abnormalities differ between the three ADHD age groups, especially when compared between child/adolescent and adults. Overall, the current work highlighted the dynamic properties of the brain captured through sliding window correlations. Furthermore, given the dFNC differences among the three groups, our work provides new insights into the mechanism subserved by age difference in the pathophysiology of ADHD and may set the grounds for future case-control studies in the individual age groups, as well as serving as a guide in the development of treatment strategies to target these specific networks in each age group.

## Data Availability Statement

The original contributions presented in the study are included in the article/Supplementary Material, further inquiries can be directed to the corresponding author/s.

## Ethics Statement

Ethical review and approval was not required for the study on human participants in accordance with the local legislation and institutional requirements. Written informed consent to participate in this study was provided by the participants’ legal guardian/next of kin.

## Author Contributions

EA, BK-B, and BB designed the study. EA organized the data and performed the analysis. EA, BK-B, and PW performed the statistical analysis. EA, BK-B, BB, HY, and SV reviewed the results. EA, BK-B, HY, and BN worked on the figures. EA and BK-B wrote the manuscript. All authors read, contributed to the revision of the manuscript, and approved the submitted version.

## Conflict of Interest

The authors declare that the research was conducted in the absence of any commercial or financial relationships that could be construed as a potential conflict of interest.

## Publisher’s Note

All claims expressed in this article are solely those of the authors and do not necessarily represent those of their affiliated organizations, or those of the publisher, the editors and the reviewers. Any product that may be evaluated in this article, or claim that may be made by its manufacturer, is not guaranteed or endorsed by the publisher.

## Footnotes

1
http://mialab.mrn.org/software/gift/
