# Supplementary material for: Differences in Disrupted Dynamic Functional Network Connectivity Among Children, Adolescents, and Adults With Attention Deficit/Hyperactivity Disorder: A Resting-State fMRI Study
Source: Front Hum Neurosci. 2021 Oct 5;15:697696. doi: 10.3389/fnhum.2021.697696 (PMC8523792; doi:10.3389/fnhum.2021.697696)
Supplement: Supplementary file 1 [file Data_Sheet_1.PDF]

Supplementary Material

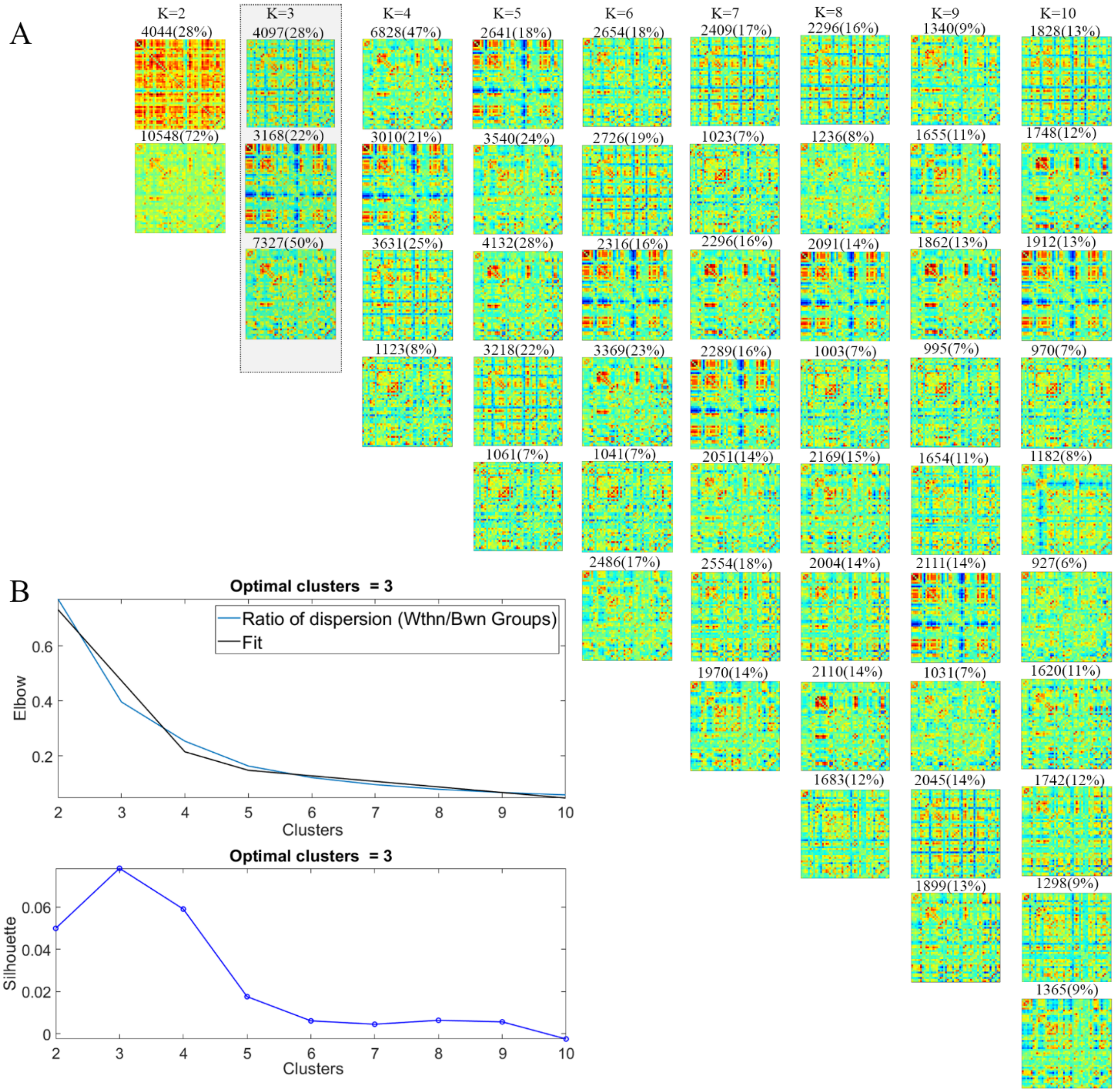

Supplementary Figure 1: Cluster centroids for k = 2 to 10.Cluster centroids from K means clustering (K=2-10). (A) Based on the selected Resting-state networks (RSNs), K-means algorithm was applied to subject windows with 500 repetitions for each K. The total number and percentage of occurrences are shown above each cluster centroid. S1 –S10 represents the various cluster states. The grey rectangle indicates the clustering results presented in this study. K=3 was selected as determined by the elbow criterion in search for optimal K-value. (B) Optimal K obtained from the elbow criterion and Silhouette for the dFNC analysis.

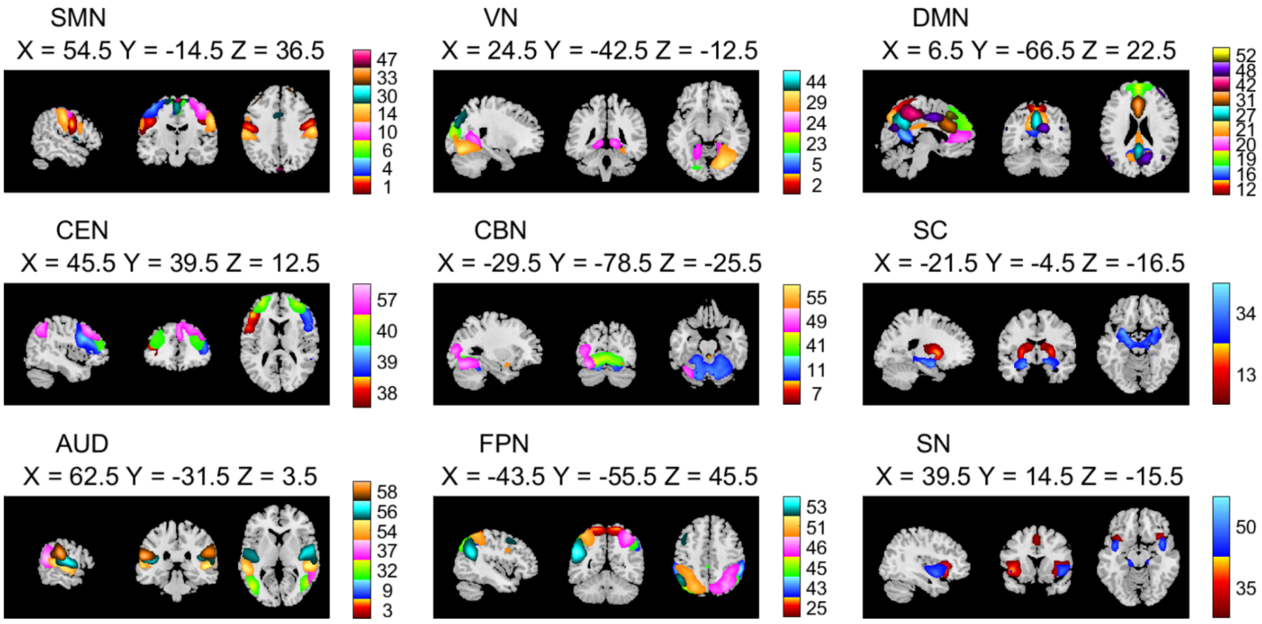

Supplementary Figure 2: Identified ICNs grouped according to their functional and anatomical properties. Each color in the composite map matches a different ICN.

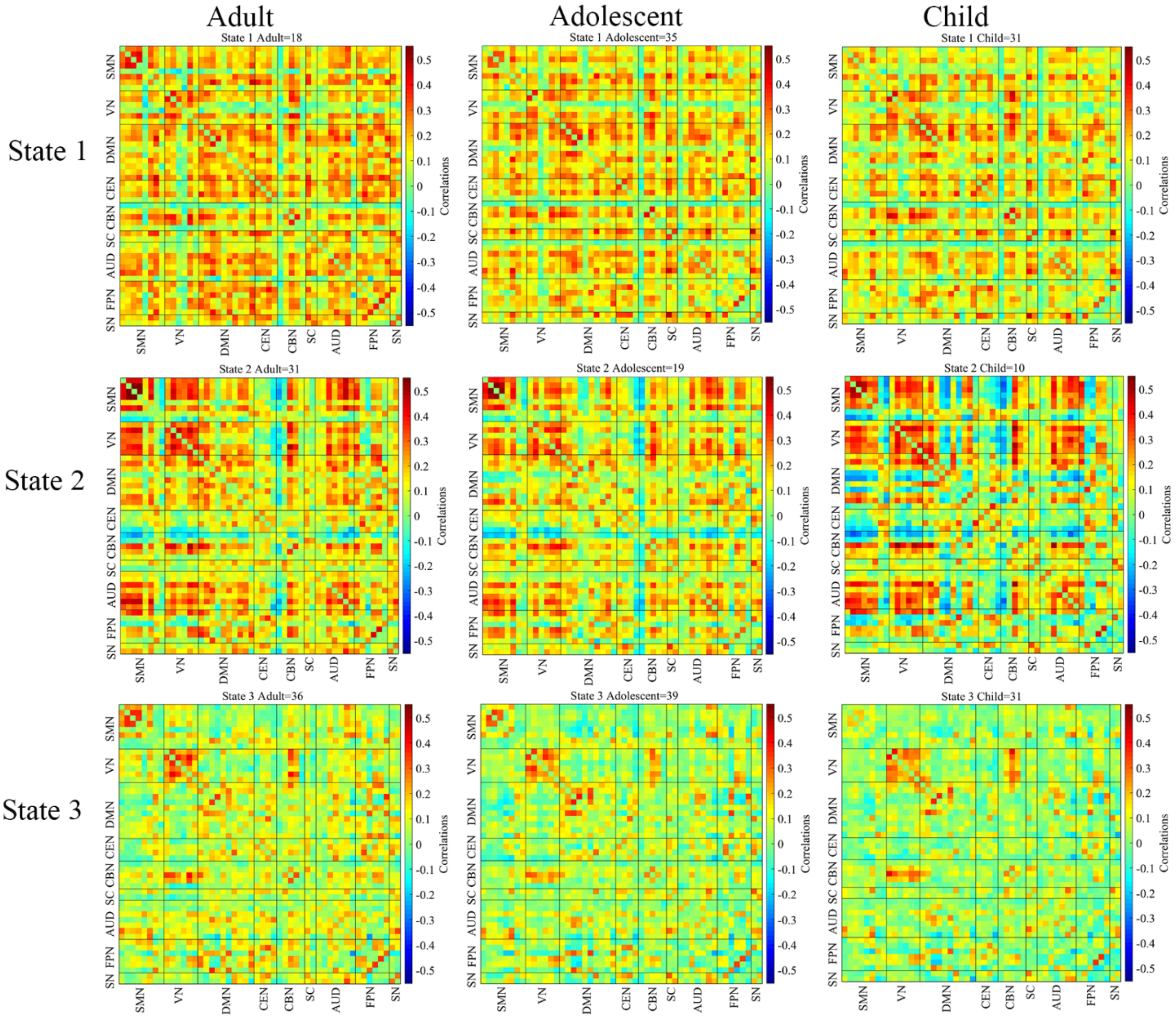

Supplementary Figure 3: Connectivity patterns of the three cluster states in each of the three age groups. The group specific centroid for the three groups across all states are shown in each column with the counts of subject having at least a window shown respectively. Connectivity patterns are generally similar between the three groups across the three cluster states.

Supplementary Table 1. Selected Independent components and their respective brain regions.

Independent Component (IC) Number	Brain Region
1	Postcentral_R
2	Calcarine_L
3	Temporal_Pole_Sup_R
4	Precentral_L
5	Cuneus_L
7	Vermis_10
9	Temporal_Sup_Pole_L
10	Precentral_R
11	Vermis_6
12	Precuneus_R
13	Putamen_R
14	Postcentral_R
16	Precuneus_L
19	Frontal_Sup_Medial_L
20	Cingulum_Ant_L
21	Precuneus_L
23	Occipital_Mid_L
24	Lingual_L

25	Parietal_Sup_R
27	Precuneus_L
29	Fusiform_R
30	Supp_Motor_Area_L
31	Cingulum_Ant_L
32	Temporal_Mid_L
33	Supp_Motor_Area_L
34	Amygdala_L
35	Insula_R
37	Temporal_Mid_R
38	Frontal_Inf_Tri_L
39	Frontal_Inf_Oper_R
40	Frontal_Mid_L
41	Cerebelum_6_L
42	Cingulum_Mid_L
43	SupraMarginal_R
44	Occipital_Mid_L
45	Angular_L
46	Parietal_Sup_R
47	Supp_Motor_Area_L

48	Cingulum_Mid_L
49	Cerebelum_Crus1_L
50	Insula_R
51	Parietal_Sup_L
52	Frontal_Sup_Medial_L
53	Angular_L
54	Temporal_Mid_R
55	Vermis_3
56	Temporal_Sup_R
57	Frontal_Sup_R
58	Temporal_Sup_L
